# Association between dietary patterns and cognitive ability in Chinese children aged 10–15 years: evidence from the 2010 China Family Panel Studies

**DOI:** 10.1186/s12889-021-12209-2

**Published:** 2021-12-04

**Authors:** Tiantian Wang, Shiyi Cao, Dandan Li, Fan Chen, Qingqing Jiang, Jing Zeng

**Affiliations:** 1 School of Public Health, Wuhan University of Science and Technology, Wuhan, 430065 Hubei China; 2 School of Public Health, Tongji Medical College, Huazhong University of Science and Technology, Wuhan, 430030 China; 3 Department of Tuberculosis Control, Wuhan Pulmonary Hospital, Wuhan, 430030 Hubei China

**Keywords:** Children, Dietary pattern, Cognitive ability, China family panel studies

## Abstract

**Background:**

Limited information is available concerning the association between dietary patterns and cognitive ability during adolescence, especially in regards to the epidemiological studies in China. Therefore, this study aimed to analyze the association between dietary patterns and cognitive ability in Chinese children aged 10–15 years.

**Methods:**

The dietary information, cognitive ability and sociodemographic data of 2029 children were retrieved from the 2010 China Family Panel Studies. Dietary patterns were assessed by principal component analysis. Ordinal logistic regression models were used to determine the association between dietary patterns and cognitive ability in these children.

**Results:**

Three dietary patterns were identified, namely, ‘High protein’, ‘High fat’ and ‘High salt-oil’. Following adjustment for gender, age, nationality, household registration, school type, parental education level, family learning environment, annual household income and family size, we found that an increase in ‘High protein’ pattern score was significantly associated with higher mathematics test scores (OR = 1.62, CI: 1.23 ~ 2.15; *P* = 0.001), but not with vocabulary test scores (OR = 1.21, CI: 0.93 ~ 1.58; *P* = 0.149). On the contrary, an increase in ‘High fat’ pattern score was significantly associated with lower scores of mathematics (OR = 0.76, CI: 0.59 ~ 0.98; *P* = 0.031) and vocabulary (OR = 0.77, CI: 0.61 ~ 0.97; *P* = 0.029) tests. However, there was no significant association between ‘High salt-oil’ pattern and the scores of mathematics (OR = 0.99, CI: 0.77 ~ 1.27; *P* = 0.915) and vocabulary (OR = 0.93, CI: 0.73 ~ 1.18; *P* = 0.544) tests.

**Conclusion:**

The findings of this study demonstrated that ‘High protein’ pattern was positively associated with cognitive ability in Chinese children, while ‘High fat’ pattern exhibited a negative association.

**Supplementary Information:**

The online version contains supplementary material available at 10.1186/s12889-021-12209-2.

## Background

The cognitive ability of children has always been a key focus of public health researchers. Children’s school performance usually affects their future education, which ultimately determines their socio-economic status. In turn, education is closely associated with health and healthy behavior [1]. Hence, more attention should be paid to the factors that influence academic achievement during childhood and adolescence.

Adolescence is a key period of brain and cognitive development, mainly due to its various developmental stages regulated by some common and independent biological processes [2]. Nutrition is one of the most essential and changeable environmental factor that can affect brain development, cognition ability and academic performance. Several studies from the United States, Australia, Fenland and the United Kingdom examined the effects of healthy dietary consumption on cognitive function in both children and adolescents, and the results showed that there was a positive association between healthier foods (e.g., whole grains [3–5], fishes [5–8], fruits and/or vegetables [9, 10]) and cognitive function. However, less-healthy snacks [8–12], sugar-sweetened beverages [11–13], chocolates [13], pizza and hot dogs [13] and red/processed meats [5] were inversely associated with cognitive function. Besides, mushrooms and nuts [14], dietary fiber [15] and regular meals [13, 16] were associated with improved cognitive performance. Children with malnutrition or insufficient nutrient intake tended to have learning restraints and developmental disabilities compared to those who had an adequate nutrient uptake [17, 18], and the malnourished children often exhibited lower academic performance in school than well-nourished children [19].

Generally, people consume a variety of foods rather than only one food [20]. The evaluation of a single food or nutrient often ignores their complex interactions, which may not reflect the total diet consumed by an individual [20, 21]. Therefore, it is important to assess the diet as a whole. In the principal components analysis (PCA), complex diet and multiple food groups are taking into account for pattern classification, instead of individual nutrients, specific foods or food groups. Besides, dietary pattern can reflect individual’s food preferences [22]; therefore, dietary pattern analysis may add more information to reflect the complexity of dietary intake and provide new insights into the whole foods diet. In the past decades, the dietary pattern of most Chinese children and adolescents was mainly consisted of cereal and plant-based foods, and then gradually changed into a Western pattern dominated by dessert, fast food, animal-based food in recent years [23]. Additionally, pickled foods and fried foods are still preferred in some regions of China.

Concerning the relationships between diets and cognition, more and more researchers began to investigate the effects of dietary patterns on cognitive functions. A previous study [24] showed that the ‘Snacky’ pattern (potatoes and other starchy roots, salty snacks, and sugar products) was negatively associated with the scales of cognitive ability in a mother-child cohort, while the ‘Western’ and the ‘Mediterranean’ patterns were not associated with child neurodevelopmental scales. Henriksson et al. [25] assessed the diet quality index, ideal diet score and Mediterranean diet score of dietary patterns, and the results suggested that healthier dietary patterns, as indicated by higher diet quality index and ideal diet score, were positively associated with attention capacity in adolescence. Nurliyana et al. [26] identified four major dietary patterns among adolescents, which were ‘refined-grain’ pattern, ‘snack-food’ pattern, ‘plant-based food’ pattern and ‘high-energy food’ pattern, and found that ‘high-energy food’ pattern was associated with lower scores on general cognitive ability. Another prospective cohort study [27] reported that higher intake of Western foods at age 14 was associated with diminished cognitive performance 3 years later, at 17 years old. Northstone et al. [28] examined the association between dietary patterns in childhood and intelligence quotient (IQ) later in life, and found that the ‘processed’ (high fat and sugar content) pattern of diet at 3 years old was negatively associated with IQ assessed at 8.5 years of age.

Most of the existing studies have focused on the relationship between dietary pattern and cognition in the elderly, and only few studies have investigated on both children and adolescents. Although the above-mentioned studies reported the associations between dietary pattern and cognitive ability during childhood and adolescence [24–28], the data available on Asia is still extremely limited, especially on Chinese children and adolescents. Therefore, in this study, we aimed to investigate the associations between dietary patterns (obtained by PCA) and cognitive ability in Chinese children aged 10–15 years.

## Methods

### Study population

In this cross-sectional study, the dietary information, cognitive ability and sociodemographic data of 2029 children aged 10–15 years were retrieved from the 2010 China Family Panel Studies (CFPS). The CFPS was carried out at Peking University. It is a nearly nationwide, comprehensive, longitudinal social survey that intends to serve research needs regarding a large variety of social phenomena in contemporary China. In that survey, the participants were recruited form 25 provinces / municipalities / autonomous regions, and the questionnaires included diverse communities, families, adults and children, which could reflect the changes of society, economy, population, education and health [29].

**Fig. 1:**
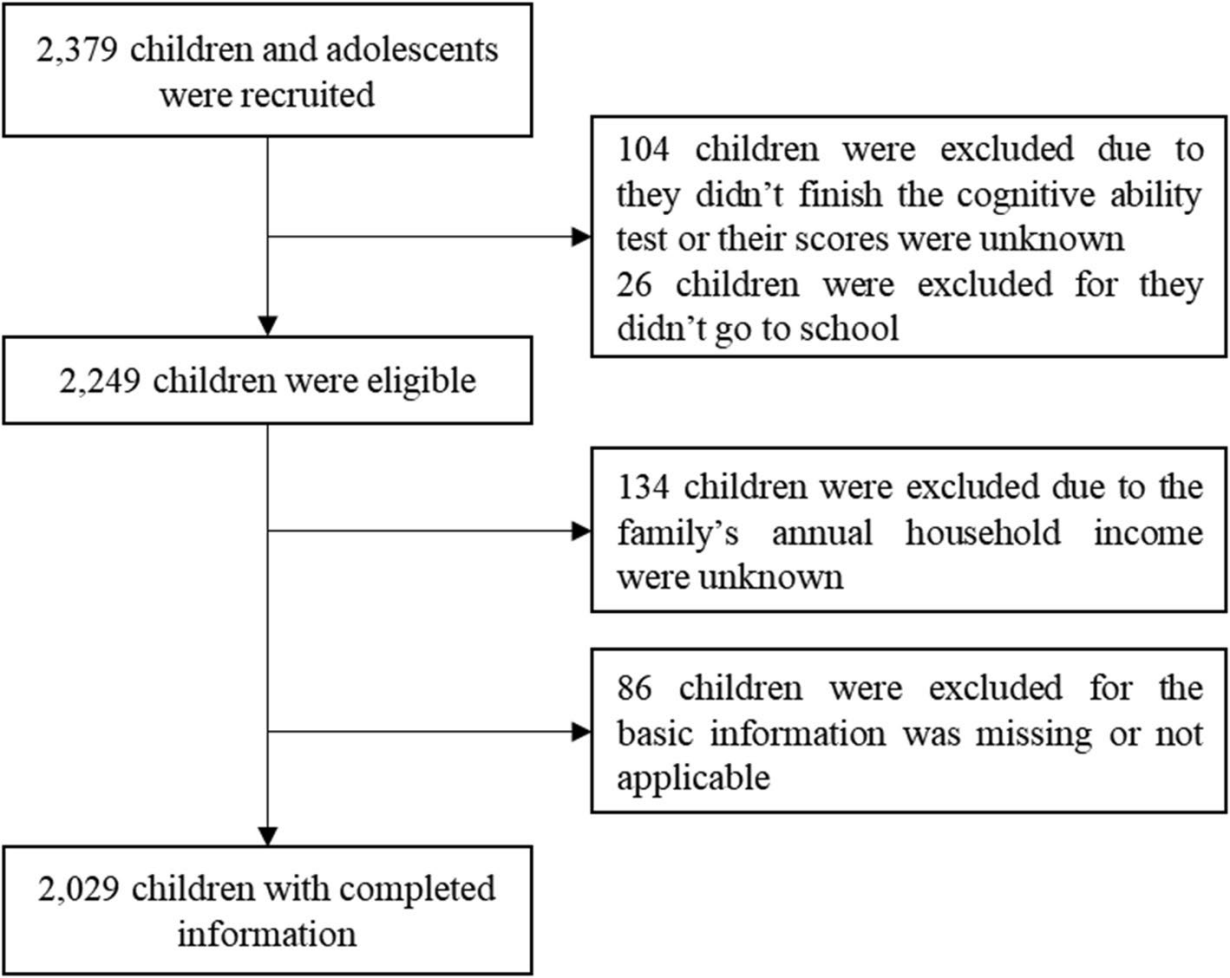
Flow diagram of subject recruitment

In the present study, we selected children aged 10–15 years as target samples. After matching and screening against children, family and community databases, we excluded the individuals whose index values were unknown, rejected, not applicable and missing. Finally, 2029 children with complete information were recruited in the analyses, and the participation rate was 85.29%. The flow diagram of subject recruitment is shown in Fig. 1. The children were approximately divided by gender (49.3% boys vs. 50.7% girls).

### Classification of dietary patterns

The dietary patterns were assessed using a food frequency questionnaire (FFQ) administered by trained investigators during face-to-face interview. This FFQ was adapted from a simplified food frequency questionnaire with good reliability and validity [30, 31]. The foods were classified into 8 categories: meat, aquatic products, fresh vegetables and fruits, milk and dairy products, beans and bean products, eggs, pickled food, and puffed and fried food. More details can be found in Supplementary File 1. All participants were exposed to the following questions: (i) whether you had eaten these eight groups of foods during the last 3 months, and (ii) the average frequency of foods intake per week in the past 3 months. Responses to the food intake frequency were ‘never’, ‘less than once’, ‘2 times’, and ‘more than 2 times’ per week in the last month. If the answer was ‘never’ or ‘not applicable’ and less than once per week, it was recorded as 0 and 1, respectively. Two times or more than 2 times per week were recorded according to their respective times. PCA method was used to identify major dietary patterns, while factor analysis was conducted to assess the frequency of food groups from FFQ [32]. The analysis was limited by factors with an eigenvalue of > 1 and a standardized loading of ≥0.40. Variance rotation was then employed to improve the separation of the factors. Finally, the factor scores were divided into four quartile (Q) groups, in which Q1 and Q4 represented the lowest and highest levels of dietary pattern scores, respectively.

### Evaluation of cognitive ability

In the CFPS 2010, all participants aged 10 and older performed the cognitive ability tests (including vocabulary and mathematics tests) by themselves after receiving information collection from individual self-answer questionnaire. The theoretical basis of the CFPS 2010 cognitive ability tests was the design of Guttman Scale in psychometry, which showed good reliability and validity [33]. The American psychologist Cattell has divided cognitive ability into two components: fluid intelligence and crystalized intelligence [34]. Fluid intelligence is on the basis of neurophysiological development, such as perception and memory. Crystal intelligence refers to the skills acquired through the accumulation of acquired knowledge and experience, such as vocabulary, calculation, speech understanding and common sense. The CFPS 2010 applied both vocabulary and mathematics tests as measurement tools to collect the crystal intelligence scores of study participants [29, 35].

The vocabulary test was consisted of 34 Chinese characters drawn from the language textbooks used in primary and secondary schools and sorted in ascending order of difficulty. This test seeks to measure one’s vocabulary by how difficult he or she can recognizes a character. To make the test more efficient, all survey respondents were assigned to one of three entry points, based on their self-reported highest level of education. The respondents were asked to recognize the increasingly difficult characters one by one until they failed to recognize three consecutive characters. The final test score was based on the rank order of the last character recognized by each respondent, ranging from 0 (lowest) to 34 (highest). Lastly, the test scores were categorized into four quartile (Q) groups: 0 ~ 17 (Q1), 18 ~ 22 (Q2), 23 ~ 26 (Q3) and 27 ~ 34 (Q4).

The mathematics test was consisted of 24 mathematical questions. The procedures of mathematics test were similar to vocabulary test. The mathematics test scores were categorized based on the same rank-order rule as that in the vocabulary test, which recorded from 0 (lowest) to 24 (highest) [36]. The mathematics test scores were also categorized into four quartile (Q) groups: 0 ~ 8 (Q1), 9 ~ 11 (Q2), 12 ~ 14 (Q3) and 15 ~ 24 (Q4). In the statistical analysis, both mathematics and vocabulary test scores were used as continuous variables (raw scores) and ordinal categorical variables (Q1-Q4).

### Covariates

Sociodemographic and family characteristics were considered as covariates, including gender, age, nationality, household registration, school type, parental educational level, family learning environment, annual household income and family size [37, 38]. Nationality was classified into two categories: Han nationality and minority nationality. Household registration included urban and countryside. Parental educational levels were categorized into three groups: (a) low (completed primary school or lower); (b) medium (completed junior middle school but did not undergo the tertiary entrance exam); and (c) high (took a tertiary entrance exam or higher). Family learning environment was classified as (a) good, (b) neutrality, and (c) bad. Annual household income (per capital) was divided into three groups: <3500RMB, 3500 –7000RMB, and > 7000RMB. Family size included 3 – 6 persons and 7 – 14 persons. The data of family characteristics were obtained from children, family and community questionnaires.

### Statistical analysis

The data were initially analyzed to generate descriptive statistics. PCA with varimax rotation was used to categorize the standardized food items. This method has been described in detail elsewhere [10]. Foods with factor loadings above 0.4 were thought to be strongly associated with the component, and were considered as the most informative variable for describing the dietary patterns. Labels were given to different components, even though these did not perfectly describe each underlying pattern. Indeed, they facilitated the reporting and discussion of results. Cognitive ability was assessed using both mathematics and vocabulary tests in the 2010 CFPS. Firstly, we analyzed the association between the covariates and cognitive ability of children using the chi-square test. Then, ordinal logistic regression was used to determine the associations among covariates, dietary patterns and cognitive ability. Finally, we constructed three models using the ordinal logistic regression method, and focused on how dietary patterns influence cognitive ability, independent of children’s basic demographic characteristics, children school type, their parents’ educational level and family factors. In Model I, we adjusted for gender, age, nationality and household registration to ensure that the outcomes were independent of the common characteristics of children and adolescents. In Model II, we further adjusted for school types and parents’ educational level. In Model III, apart from the variables included in Models I and II, we additionally adjusted for family characteristics, family learning environment, annual household income and family size. All analyses were carried out using STATA 13.0. *P*-value < 0.05 was considered statistically significant.

## Results

### Characteristics of the study subjects

The characteristics of each participant are shown in Table 1. Approximately half of the participants were girls (50.7%, *n* = 1001), and most of them were Han nationality (88.4%, *n* = 1794). Nearly 60.0% of the participants (*n* = 1228) came from the countryside.

**Table 1:** Descriptive statistics of 2029 participants

	Number	Percentage (%)
**Gender**		
Girl	1001	50.7
Boy	1028	49.3
**Age**		
10	342	16.9
11	348	17.2
12	325	16.0
13	320	15.8
14	334	16.5
15	360	17.7
**Nationality**		
Minority nationality	235	11.6
Han nationality	1794	88.4
**Household registration**		
Countryside	1228	60.5
Urban	801	39.5
**School**		
Common school	1940	95.6
Key school	89	4.4
**Father education level** ^**a**^		
Low	935	46.1
Medium	973	48.0
High	121	6.0
**Mother education level** ^**a**^		
Low	1245	61.4
Medium	693	34.2
High	91	4.5
**Family learning environment**		
Bad	195	9.61
Neutrality	877	43.22
Good	957	47.17
**Annual household income (per person)**		
< 3500RMB	680	33.5
3500 – 7000RMB	696	34.3
> 7000RMB	653	32.2
**Family size**		
3 – 6	1822	89.8
7 – 14	207	10.2
**Mathematics test (score)**^**b**^		
0–8	536	26.4
9–11	476	23.5
12–14	536	26.4
14–24	481	23.7
**Vocabulary test (score)**^**c**^		
0–17	486	24.0
18–22	504	24.8
23–26	462	22.8
27–34	577	28.4

^a^Education level: low (completed primary school or lower); medium (completed junior middle school but did not undergo the tertiary entrance exam); and high (had taken the tertiary entrance exam or higher)

^b^The scores of mathematics test ranged from 0 to 24

^c^The scores of vocabulary test ranged from 0 to 34

### Dietary patterns of the study participants

The factor loadings of different dietary patterns are presented in Table 2. Three dietary patterns were identified and labeled as ‘High protein’, ‘High fat’ and ‘High salt-oil’. Foods that loaded highly on the ‘High protein’ dietary pattern were milk, dairy products, eggs, beans and bean products. Meat and aquatic products were loaded highly on the ‘High fat’ dietary pattern. Pickled food as well as puffed and fried food were loaded highly on the ‘High salt-oil’ dietary pattern.

**Table 2:** Factor loadings of the three dietary patterns^a^

Food group	‘High protein’ ^b^	‘High fat’ ^c^	‘High salt-oil’ ^d^
Meat	0.0261	**0.6531**^**a**^	0.0189
Aquatic products	−0.0321	**0.6523**^**a**^	−0.1367
Vegetables and fruits	0.0031	0.3687	0.3287
Milk and dairy products	**0.5713**^**a**^	0.0485	−0.0633
Bean and bean products	**0.5605**^**a**^	−0.0514	0.0791
Eggs	**0.5851**^**a**^	−0.0076	−0.0528
Pickled food	−0.0547	−0.0751	**0.7632**^**a**^
Puffed and fried food	0.1111	0.0375	**0.5266**^**a**^

^a^Dietary pattern factor loadings ≥0.4

^b^‘High protein’ dietary pattern (milk and dairy products, bean and bean products, and eggs)

^c^‘High fat’ dietary pattern (meat and aquatic products)

^d^‘High salt-oil’ dietary pattern (pickled food, and puffed and fried food)

### Influencing factors of cognitive ability: results from chi-square test

The chi-square test results for the association between children’s cognitive ability and its influencing factors are demonstrated in Table 3. In the cognitive ability test, there were statistically significant relationships between mathematics scores and all variables (*P* < 0.05), except for gender (*P* > 0.05). Similarly, there were statistically significant relationships between vocabulary scores and all variables (*P* < 0.05).

**Table 3:** Influencing factors for the cognitive ability of children aged 10–15 years

Variables	Mathematics test scores ^a^				*χ*^2^	*P* value	Vocabulary test scores ^a^				*χ*^2^	*P* value
	Q1 [0–8] n (%)	Q2 [9–11] n (%)	Q3 [12–14] n (%)	Q4 [15–24] n (%)			Q1 [0–17] n (%)	Q2 [18–22] n (%)	Q3 [23–26] n (%)	Q4 [27–34] n (%)		
n	536	476	536	481			486	504	462	577		
**Gender**												
Girl	271 (50.6)	226 (47.5)	270 (50.4)	234 (48.6)	1.299	0.729	208 (42.8)	237 (47.0)	233 (50.4)	323 (56.0)	19.798	< 0.001
Boy	265 (49.4)	250 (52.5)	266 (49.6)	247 (51.4)			278 (57.2)	267 (53.0)	229 (49.6)	254 (44.0)		
**Age**												
10	224 (41.8)	106 (22.3)	9 (1.7)	3 (0.6)	1.4e+ 03	< 0.001	180 (37.0)	97 (19.3)	43 (9.3)	22 (3.8)	485.985	< 0.001
11	147 (27.4)	161 (33.8)	36 (6.7)	4 (0.8)			125 (25.7)	107 (21.2)	76 (16.5)	40 (6.9)		
12	87 (16.2)	115 (24.2)	100 (18.7)	23 (4.8)			80 (16.5)	98 (19.4)	78 (16.9)	69 (12.0)		
13	46 (8.6)	60 (12.6)	161 (30.0)	53 (11.0)			49 (10.1)	77 (15.3)	87 (18.8)	107 (18.5)		
14	18 (3.4)	22 (4.6)	144 (26.9)	150 (31.2)			22 (4.5)	66 (13.1)	99 (21.4)	147 (25.5)		
15	14 (2.6)	12 (2.5)	86 (16.0)	248 (51.6)			30 (6.2)	59 (11.7)	79 (17.1)	192 (33.3)		
**Nationality**												
Minority nationality	439 (81.9)	412 (86.6)	495 (92.4)	448 (93.1)	42.395	< 0.001	104 (21.4)	65 (12.9)	36 (7.8)	30 (5.2)	76.023	< 0.001
Han nationality	97(18.1)	64(13.4)	41(7.6)	33 (6.9)			382 (78.6)	439 (87.1)	426 (92.2)	547 (94.8)		
**Household registration**												
Countryside	386 (72.0)	277 (58.2)	313 (58.4)	252 (52.4)	45.037	< 0.001	340 (70.0)	327 (64.9)	272 (58.9)	289 (50.1)	48.945	< 0.001
Urban	150 (28.0)	199 (41.8)	223 (41.6)	229 (47.6)			146 (30.0)	177 (35.1)	190 (41.1)	288 (49.9)		
**School**												
Common school	525 (98.0)	466 (97.9)	505 (94.2)	444 (92.3)	27.921	< 0.001	476 (97.9)	495 (98.2)	447 (96.8)	522 (90.5)	52.271	< 0.001
Key school	11 (2.0)	10 (2.1)	31 (5.8)	37 (7.7)			10 (2.1)	9 (1.8)	15 (3.2)	55 (9.5)		
**Family learning environment**												
Bad	79 (40.5)	36 (18.5)	58 (29.7)	22 (11.3)	56.343	< 0.001	68 (34.9)	50 (25.6)	49 (25.1)	28 (14.4)	61.321	< 0.001
Neutrality	261 (29.8)	203 (23.2)	221 (25.2)	192 (21.9)			240 (27.4)	238 (27.1)	176 (20.1)	223 (25.4)		
Good	196 (20.5)	237 (24.8)	257 (26.9)	267 (27.9)			178 (18.6)	216 (22.6)	237 (24.8)	326 (34.1)		
**Father education level**												
Low	309 (57.7)	211 (44.3)	244 (45.5)	171 (35.5)	90.999	< 0.001	286 (58.9)	246(48.8)	207 (44.8)	196 (34.0)	116.108	< 0.001
Medium	211 (44.3)	237 (49.8)	260 (48.5)	265 (55.1)			186 (38.3)	240(47.6)	227 (49.1)	320 (55.5)		
High	16 (3.0)	28 (5.9)	32 (6.0)	45 (9.4)			14 (2.9)	18(3.6)	28 (6.1)	61 (10.6)		
**Mother education level**												
Low	405 (75.6)	276 (58.0)	305 (56.9)	259 (53.8)	119.648	< 0.001	361 (74.3)	337 (66.9)	271 (58.7)	276 (47.8)	156.641	< 0.001
Medium	116 (21.6)	181 (38.0)	209 (39.0)	187 (38.9)			116 (23.9)	155 (30.8)	170 (36.8)	252 (43.7)		
High	15 (2.8)	19 (4.0)	22 (4.1)	35 (7.3)			9 (1.9)	12 (2.4)	21 (4.6)	49 (8.5)		
**Annual household income (per person)**												
< 3500RMB	244 (45.5)	143 (30.0)	155 (32.6)	122 (25.4)	67.300	< 0.001	208 (42.8)	182 (36.1)	145 (31.4)	145 (25.1)	55.669	< 0.001
3500 7000 RMB	167 (31.2)	178 (37.4)	190 (35.5)	153 (31.8)			163 (33.5)	176 (34.9)	158 (34.2)	191 (33.1)		
> 7000 RMB	125 (23.3)	155 (32.6)	175 (32.7)	206 (42.8)			115 (23.7)	146 (29.0)	159 (34.4)	241 (41.8)		
**Family size**												
3 – 6	455 (84.9)	425 (89.3)	487 (90.9)	455 (94.6)	26.978	< 0.001	412 (84.8)	445 (88.3)	423 (91.6)	542 (93.9)	26.975	< 0.001
7 –14	81 (15.1)	51 (10.7)	49 (9.1)	26 (5.4)			74 (15.2)	59 (11.7)	39 (8.4)	35 (6.1)		

^a^*Q* Quartile; Q1, Q2, Q3 and Q4 represent 0–25%, 25–50%, 50–75% and 75–100%, respectively

### Influencing factors of cognitive ability: results from ordinal-logistic regression

As is shown in Table 4, there was a positive relationship between ‘High protein’ dietary pattern and mathematics and vocabulary test scores. The results indicated that a 1-unit increase in ‘High protein’ dietary pattern scores was associated with a 1.28-fold (CI: 1.21 ~ 1.35) increase in children’s mathematics test scores or a 1.25-fold (CI: 1.18 ~ 1.32) increase in children’s vocabulary test scores. In contrast, there were significant inverse relationships between ‘High fat’ (OR = 0.89, CI: 0.83 ~ 0.96; *P* = 0.002) and ‘High salt-oil’ (OR = 0.91, CI:0.84 ~ 0.98; *P* = 0.012) dietary patterns and vocabulary test scores. For the influencing factors associated with children’s cognitive ability, the results of Ordinal-Logistic regression were similar to those of chi-square test.

**Table 4:** Ordinal logistic regression for the association between cognitive ability test scores and covariates in children aged 10–15 years obtained from the 2010 CFPS

Variables	Mathematics			Vocabulary		
	*OR*	*P* value	95%CI	*OR*	*P* value	95%CI
‘High protein’ dietary patterns	1.28	< 0.001	(1.21; 1.35)	1.25	< 0.001	(1.18; 1.32)
‘High fat’ dietary pattern	0.96	0.287	(0.90; 1.03)	0.89	0.002	(0.83; 0.96)
‘High salt-oil’ dietary pattern	0.96	0.503	(0.90; 1.05)	0.91	0.012	(0.84; 0.98)
**Gender**						
Girl			0			0
Boy	1.02	0.763	(0.88; 1.20)	0.70	< 0.001	(0.60; 0.82)
**Age**						
10 ~ 15	2.93	< 0.001	(2.73; 3.13)	1.73	< 0.001	(1.64; 1.82)
**Nationality**						
Minority nationality			0			0
Han nationality	2.23	< 0.001	(1.74;2.86)	2.97	< 0.001	(2.31; 3.83)
**Household registration**						
Countryside			0			0
Urban	1.65	< 0.001	(1.40;1.93)	1.77	< 0.001	(1.51; 2.08)
**School**						
Common school			0			0
Key school	2.68	< 0.001	(1.82; 3.94)	4.08	< 0.001	(2.66; 6.27)
**Family learning environment**						
Bad			0			0
Neutrality	1.56	0.002	(1.18; 2.07)	1.46	0.007	(1.11; 1.93)
Good	2.26	< 0.001	(1.71; 2.99)	2.38	< 0.001	(1.81; 3.14)
**Father education level**						
Low			0			0
Medium	1.62	< 0.001	(1.38; 1.91)	1.79	< 0.001	(1.52; 2.10)
High	2.56	< 0.001	(1.81; 3.60)	3.72	< 0.001	(2.60; 5.31)
**Mother education level**						
Low			0			0
Medium	1.71	< 0.001	(1.45; 2.02)	2.00	< 0.001	(1.69; 2.37)
High	2.35	< 0.001	(1.59; 3.47)	4.11	< 0.001	(2.73; 6.16)
**Annual household income (per person)**						
< 3500RMB			0			0
3500 7000 RMB	1.46	< 0.001	(1.20; 1.76)	1.41	< 0.001	(1.16; 1.70)
> 7000 RMB	1.46	< 0.001	(1.71; 2.53)	2.09	< 0.001	(1.72; 2.54)
**Family size**						
7 14			0			0
3 6	1.97	< 0.001	(1.52; 2.55)	1.98	< 0.001	(1.53; 2.56)

### Association between dietary patterns and cognitive ability

The relationships between cognitive ability and dietary patterns (both as continuous variables and quartile) in the three models are demonstrated in Table 5. An increase in ‘High protein’ dietary pattern score (continuous variable) was associated with higher mathematics test scores, and the results were similar for crude model (OR = 1.29, CI: 1.21 ~ 1.37; *P* < 0.001) and fully adjusted model (OR = 1.15, CI: 1.07 ~ 1.23; *P* < 0.001). The relationships between ‘High protein’ dietary pattern and vocabulary test scores were statistically significant in Models I and II, but not in Model III (OR = 1.06, CI: 0.99 ~ 1.13; *P* = 0.076). Meanwhile, an increase in ‘High fat’ dietary pattern score (continuous variable) was significantly associated with lower mathematics test scores in the crude model (OR = 0.89, CI: 0.82 ~ 0.96; *P* = 0.004) and vocabulary tests in the three models (OR = 0.88, CI: 0.82 ~ 0.96; *P* = 0.002 in model III). However, children with increased scores of ‘High salt-oil’ dietary pattern (continuous variable) tended to have lower scores of vocabulary test in Model I (OR = 0.90, CI: 0.83 ~ 0.98; *P* = 0.012).

**Table 5:** Ordinal logistic regression models describing the association between cognitive ability test scores and dietary patterns in children aged 10–15 years (both as continuous variables and quartiles)

Test	Dietary pattern	Quartile	Model I ^a^			Model II ^b^			Model III ^c^		
			*OR*	*P* value	95%CI	*OR*	*P* value	95%CI	*OR*	*P* value	95%CI
Mathematics	‘High protein’		1.29	< 0.001	(1.21;1.37)	1.19	< 0.001	(1.11;1.27)	1.15	< 0.001	(1.07;1.23)
	‘High fat’		0.89	0.004	(0.82;0.96)	0.93	0.090	(0.86;1.01)	0.94	0.136	(0.86;1.02)
	‘High salt-oil’		0.94	0.182	(0.87;1.03)	1.00	0.955	(0.92;1.09)	1.03	0.568	(0.94;1.12)
Vocabulary	‘High protein’		1.19	< 0.001	(1.12;1.26)	1.10	0.005	(1.03;1.17)	1.06	0.076	(0.99;1.13)
	‘High fat’		0.85	< 0.001	(0.78;0.91)	0.88	0.002	(0.82;0.95)	0.88	0.002	(0.82;0.96)
	‘High salt-oil’		0.90	0.012	(0.83;0.98)	0.95	0.238	(0.88;1.03)	0.96	0.372	(0.89;1.05)
Mathematics	‘High protein’	Q1			0			0			0
		Q2	1.20	0.151	(0.94;1.54)	1.10	0.449	(0.86;1.42)	1.06	0.664	(0.82;1.36)
		Q3	2.09	< 0.001	(1.63;2.68)	1.80	< 0.001	(1.40;2.33)	1.67	< 0.001	(1.29;2.16)
		Q4	2.47	< 0.001	(1.90;3.20)	1.84	< 0.001	(1.41;2.41)	1.62	0.001	(1.23;2.15)
	‘High fat’	Q1			0			0			0
		Q2	0.74	0.014	(0.58;0.94)	0.84	0.160	(0.66;1.07)	0.86	0.234	(0.67;1.10)
		Q3	0.85	0.182	(0.67;1.08)	0.95	0.700	(0.75;1.22)	1.00	0.995	(0.78;1.28)
		Q4	0.69	0.002	(0.54;0.87)	0.77	0.042	(0.61;0.99)	0.76	0.031	(0.59;0.98)
	‘High salt-oil’	Q1			0			0			0
		Q2	0.58	< 0.001	(0.45;0.74)	0.66	0.001	(0.52;0.85)	0.73	0.013	(0.56;0.93)
		Q3	0.55	< 0.001	(0.43;0.71)	0.68	0.003	(0.53;0.87)	0.75	0.028	(0.59;0.97)
		Q4	0.76	0.028	(0.60;0.97)	0.91	0.431	(0.71;1.16)	0.99	0.915	(0.77;1.27)
Vocabulary	‘High protein’	Q1			0			0			0
		Q2	1.18	0.170	(0.93;1.48)	1.07	0.580	(0.85;1.35)	1.03	0.776	(0.82;1.31)
		Q3	1.29	0.036	(1.02;1.63)	1.10	0.438	(0.87;1.40)	1.03	0.809	(0.81;1.31)
		Q4	1.82	< 0.001	(1.43;2.33)	1.36	0.017	(1.06;1.75)	1.21	0.149	(0.93;1.58)
	‘High fat’	Q1			0			0			0
		Q2	0.94	0.622	(0.75;1.19)	1.03	0.778	(0.82;1.31)	1.06	0.624	(0.84;1.34)
		Q3	0.85	0.165	(0.68;1.07)	0.94	0.627	(0.75;1.19)	0.97	0.780	(0.76;1.22)
		Q4	0.69	0.002	(0.55;0.87)	0.78	0.036	(0.62;0.98)	0.77	0.029	(0.61;0.97)
	‘High salt-oil’	Q1			0			0			0
		Q2	0.65	< 0.001	(0.51;0.81)	0.75	0.016	(0.59;0.95)	0.80	0.064	(0.63;1.01)
		Q3	0.67	0.001	(0.53;0.85)	0.82	0.101	(0.65;1.04)	0.88	0.305	(0.69;1.12)
		Q4	0.75	0.014	(0.59;0.94)	0.87	0.269	(0.69;1.11)	0.93	0.544	(0.73;1.18)

^a^Model I includes gender, age, nationality, and household registration

^b^Model II includes the variables in model 1 + school type, mother education, and father education

^c^Model III includes the variables in model 2 + family education environment, family income, and family size

When dividing the ‘High protein’, ‘High fat’ and ‘High salt-oil’ dietary patterns into four quartiles, the obtained results were similar to the above-described relationships between continuous dietary pattern scores and cognitive ability outcomes.

## Discussion

In the present study, we found that children with higher scores of ‘High protein’ dietary pattern tended to be associated with better cognitive ability. However, children with higher score of ‘High fat’ dietary pattern were associated with poorer cognitive ability. Thus, it is of great importance for families to select a proper dietary pattern for children and adolescents.

### Sociodemographic characteristics and cognitive ability of children

Our findings indicated that parents with higher education and lived in a better family learning environment were related to children’s higher cognition scores. Previous studies reported that parent’s educational status could indicate 19% of the variance in children’s intelligence [37], and children whose parents had higher levels of education were more likely to achieve higher IQ scores [38, 39]. In addition, parents with higher education usually paid more attention to early childhood education, and they would cultivate their children more concertedly, thus promoting children’s cognitive development [39].

Besides, this study found that children who lived in the rural areas and a large family tended to have diminished cognitive ability. The reason might be that child poverty is common among rural communities and large families. In poor household, parents are less engaged in their children’s school performance, thus children may suffer from less educational resources and subsequently have poorer cognitive outcomes [40, 41]. Our findings of the relationship between annual household income and cognition ability also supported this view, which were consistent with studies in Australia and America [42, 43]. In our study, about half of the parents finished the elementary school, and 33.5% children whose family annual income were less than 3500RMB. Above all, the economic status and educational attainment in our country still need to be improved substantially.

### Association between dietary patterns and cognitive ability in children

Cognitive ability has been shown to be affected by a good diet (e.g., fish and milk) [8, 44] and a bad diet (e.g., French fries, hot dogs, soft drinks and red meat) [45, 46]. These findings were consistent with our results concerning the associations between dietary patterns and cognition ability in children. We found that children with ‘High protein’ dietary pattern (higher intakes of milk, dairy products, beans, bean products and eggs) often achieved higher cognitive ability test scores.

Adolescence is an important period of a child’s growth and development, and the demanding of nutrition is the highest during this period. Therefore, it is necessary to meet the intake of protein, especially high-quality protein. Milk, beans and eggs are the major sources of high-quality protein, which play an important role in the daily meals. The analysis of food consumption patterns in China has revealed that the annual consumption of milk, bean, eggs and other food is increasing, but there is still a considerable gap between the actual and recommended dietary intakes of milk, beans and eggs [47, 48]. Previous studies in Kenya and South Korea demonstrated that higher intake of milk and dairy products was associated with better academic achievement [44, 49]. Another study [50] showed that higher milk consumption could improve overall nutritional status in Korea adolescents, and the well-nourished children often performed better than the malnourished children [19]. In addition, higher consumption of meat or egg during breakfast might contribute to improved cognitive ability in youth [51].

Milk, dairy products, beans, bean products and eggs are good sources of high-quality protein. A previous study indicated that there was a positive correlation between academic performance in elementary and middle school and frequent consumption of dairy and eggs [52]. The proteins positively influence hippocampal function and childhood development of higher cognitive processes [53]. An animal research demonstrated that the tasks mediated through hippocampal function were mainly affected by protein consumption [54]. Collectively, high intake of protein was significantly related to better cognition in both children and adolescents.

In addition, this study showed that ‘High fat’ dietary pattern was associated with poorer cognitive ability, which might be attributed to the fact that children consumed a large amount of red meat or refined meat. In the recent years, the contradiction between food supply and demand in China is not only manifested by the excessive production of grain and meat, but also the shortage of milk, beans, eggs, fruits and other food products. Specifically, the consumption of meat has approached the maximum recommended intake of 75 g according to the Chinese dietary guidelines [47, 48]. A previous study [27] reported that ‘Western’ dietary pattern at the age of 14 (high intake of takeout foods, red and processed meat, soft drinks, fried and refined foods) exerted a negative impact on the cognitive performance of 17 year-old adolescents in Perth. Besides, another study reported that a high consumption of sausage, fast food, snacks and sugar sweetened beverages at the age of 3 years was related to poorer academic achievement at the age of 10 years among children [55]. A study in Malaysia found ‘high-energy’ dietary pattern (high intake of noodle, processed meat, egg and fish snacks) was associated with lower scores of general cognitive ability [26].

Red meat, fast food or snacks usually contain more saturated fatty acids, and excessive intake of saturated fatty acids may confer a negative effect on cognition ability. Some reviews suggested that higher intake of polyunsaturated fatty acids could improve brain and cognitive development [56, 57]. Polyunsaturated fatty acids are an essential factor for normal brain development as they regulate cell membrane dynamics and modulate the endocannabinoid system that, in turn, regulates neurotransmission and participates in synaptic plasticity [58]. Moreover, dietary pattern with higher saturated fat may have adverse effects on synapsin I, growth associated protein 43, hippocampal brain derived neurotrophic factor (BDNF) and BDNF-related synaptic plasticity [59, 60]. Altogether, these studies demonstrated that a dietary pattern high in meat and saturated fat or low in PUFAs was related to lower cognition and academic performance.

### Strengths and limitations

One of the strengths was that we used PCA to identify dietary patterns, making it possible to analyze their effects on cognitive ability as a whole, rather than as a single food or nutrient. In addition, the data used in this study were retrieved from a large, comprehensive and longitudinal survey with excellent quality control, and the study population was representative of the general situation of Chinese children aged 10–15 years. Furthermore, the cognitive ability test in CFPS 2010 based on the educational level of the interviewees to choose the corresponding starting point of the answer questions, and the test questions was based on primary and secondary school textbooks. The results of these tests were reliable and could as a means to determine the level of cognitive ability [33].

A limitation of this study was that, during the process of the PCA, some factors (e.g., the number of dietary patterns and food group classification) were prone to selection bias. Moreover, we only analyzed the data of CFPS 2010, which was a cross-sectional study. When adjusting for several known confounders, we acknowledged that the information on other variables (e.g., mother’s intelligence) might serve as important drivers of cognitive ability. Therefore, more longitudinal and intervention studies with larger sample size are warranted to on the explore the long-term effects of dietary patterns on cognitive ability and academic performance in both children and adolescents. Despite that, our findings could provide valuable information to help develop public health messages and interventions.

## Conclusions

In summary, ‘High protein’ dietary pattern was identified as a favorable factor for improving the cognitive ability of children, while ‘High fat’ dietary pattern could serve as a risk factor. Both childhood and adolescence are the most sensitive periods of brain development and vulnerability to nutrient deficiency. Therefore, public health policies and health promotion programs should realize the importance of targeting food intake patterns during these critical periods.

## Supplementary Information

**Additional file 1: Supplementary file 1.** Dietary patterns, food groups and their components.

## Data Availability

Publicly available databases were analyzed in this study. The databases for this study are available from the CFPS at http://www.isss.pku.edu.cn/cfps/.

## Abbreviations

PCA: Principal components analysis
IQ: Intelligence quotient
CFPS: China Family Panel Studies
FFQ: Food-frequency questionnaire
Q: Quartiles
