# Supplementary material for: Association between dietary patterns and cognitive ability in Chinese children aged 10–15 years: evidence from the 2010 China Family Panel Studies
Source: BMC Public Health. 2021 Dec 4;21:2212. doi: 10.1186/s12889-021-12209-2 (PMC8642971; doi:10.1186/s12889-021-12209-2)
Supplement: Supplementary file 1 — Additional file 1: Supplementary file 1. Dietary patterns, food groups and their components. [file 12889_2021_12209_MOESM1_ESM.doc]

**Supplementary file 1**. Dietary patterns, food groups and their components.

| **Dietary pattern** | **Food groups** | **Components** |
| --- | --- | --- |
| High protein | Bean and bean products | Legumes, tofu, soy beans, soy milk, and bean curd sticks. |
|  | Eggs | Egg, duck egg, goose egg, and quail egg. |
|  | Milk and dairy products | Milk, yaourt, yogourt, and cheese. |
| High fat | Meat | Pork, beef, mutton, lamb, rabbit, chicken, duck, goose, haslet, sausage, ham, and preserved meat. |
|  | Aquatic products | Fish, shrimp, aquid, crab, and seashell. |
| High salt-oil | Pickled food | Pickled vegetables, fermented bean curd, bean paste, canned food, pickled meat, and pickled fish. |
|  | Puffed and fried food | Chips, shrimp bars, popcorn, rice cracker, Chinese doughnut, and French fries. |
